# Improvement in quality of life and angina pectoris: 1-year follow-up of patients with refractory angina pectoris and spinal cord stimulation

**DOI:** 10.1007/s12471-020-01422-0

**Published:** 2020-05-19

**Authors:** F. E. Vervaat, A. van der Gaag, H. van Suijlekom, C. J. Botman, K. Teeuwen, I. Wijnbergen

**Affiliations:** 1 Department of Cardiology, Catharina Hospital, Eindhoven, The Netherlands; 2 Department of Anaesthesiology and Pain Management, Catharina Hospital, Eindhoven, The Netherlands

**Keywords:** Refractory angina pectoris, Spinal cord stimulation

## Abstract

**Aims:**

Spinal cord stimulation (SCS) is a treatment for patients with refractory angina pectoris (RAP) who remain symptomatic despite optimal medical therapy and without revascularisation options. Previous studies have shown that SCS improves the quality of life in this patient group and reduces the severity of the angina pectoris. The aim of this prospective, single-arm observational study is to show this effect in a single-centre cohort using a multidisciplinary team approach to the selection process, with a follow-up period of 1 year.

**Methods and results:**

Between July 2010 and March 2017, 87 patients with RAP referred to our centre received SCS. The Seattle Angina Questionnaire (SAQ) and RAND 36-Item Health Survey (RAND-36) were completed at baseline, prior to implantation, and 1 year post-implantation. After 1 year of follow-up there was a statistically significant decrease in the frequency of angina pectoris attacks from more than 4 times a day to 1–2 times a week (*p* < 0.001). The SAQ showed statistically significant improvement in four of the five dimensions: physical limitation (*p* < 0.001), angina frequency (*p* < 0.001), angina stability (*p* < 0.001) and quality of life (*p* < 0.001). The RAND-36 showed statistically significant improvement in all nine dimensions: physical functioning (*p* = 0.001), role/physical (*p* < 0.001), social functioning (*p* = 0.03), role/emotional (*p* < 0.05), bodily pain (*p* < 0.001), general health (*p* < 0.001), vitality (*p* < 0.001), mental health (*p* = 0.02) and health change (*p* < 0.001).

**Conclusion:**

This study showed a significant improvement in quality of life and reduction of angina pectoris severity after 1 year of follow-up in patients treated with SCS for RAP.

**Electronic supplementary material:**

The online version of this article (10.1007/s12471-020-01422-0) contains supplementary material, which is available to authorized users.

## What’s new?

- Improvement in quality of life and reduction in frequency of angina pectoris in patients with refractory angina pectoris and treatment with spinal cord stimulation.
- Uniform selection process with clear inclusion criteria performed by a multidisciplinary team.

## Introduction

In recent decades, the evolution of medical therapy, coronary artery bypass grafting (CABG) and percutaneous coronary interventions (PCI) has significantly reduced the morbidity and mortality in patients presenting with stable coronary artery disease (CAD). Despite all these treatment innovations, 5–10% of patients with stable CAD remain symptomatic despite optimal therapy referred to as ‘refractory angina pectoris (RAP)’. This condition is defined as a ‘chronic condition (>3 months) characterised by diffuse CAD in the presence of proven ischaemia, which is not amendable to a combination of medical therapy, angioplasty or coronary bypass surgery’ [1]. Patients with RAP are severely restricted in performing daily activities by debilitating angina complaints leading to decreased quality of life.

Spinal cord stimulation (SCS) is a treatment option that has been developed for patients with RAP to improve quality of life and reduce the frequency of angina pectoris episodes. Four possible mechanisms explaining the beneficial effects of SCS on RAP have been described: reduction of pain perception, decreased sympathetic tone, reduced myocardial oxygen demand and improved coronary microcirculatory blood flow [2, 3].

The number of trials regarding SCS in RAP published in recent years is limited. These studies showed an improvement in quality of life and a reduction in the severity of angina pectoris after SCS. However, evaluation of these studies revealed limitations which include methodological inconsistencies, heterogeneity in primary and secondary outcome measures and the inability to recruit sufficient numbers of patients to achieve significant statistical power to provide convincing and definitive conclusions [4–9].

The aim of this open, prospective, single-arm observational study, involving a multidisciplinary team during the selection process, is to show the effects of SCS on the severity of angina complaints and quality of life in a large, real-life single-centre cohort.

## Methods

### Patient selection

Patients referred to our hospital (Catharina Hospital Eindhoven, The Netherlands) with RAP were included if the following criteria were met: stable angina pectoris Canadian Cardiovascular Society (CCS) class III or IV for at least 3 months, significant CAD with no options for revascularisation (CABG and/or PCI) and optimal medical anti-angina therapy.

At the initial visit patients performed a symptom-inducing treadmill test to evaluate the effect of transcutaneous electrical nerve stimulation (TENS). Two electrodes were applied to the chest region and connected to a battery-operated TENS device (eco 2, Schwa-medico BV, Woudenberg, The Netherlands). The standard settings of the TENS device during the treadmill test were a pulse width of 200 µS (range 50–400 µS) and a frequency of 2 Hz (range 2–100 Hz). The treadmill (Enraf-Nonius BV, Rotterdam, The Netherlands) was started at 3.5 km/h, increasing the speed every 30 s to a maximum of 5.5 km/h. When the maximum pace was reached the inclination angle increased by 1% every 30 s until angina pectoris was induced. The TENS device was switched on and the amplitude turned up to 40 mA (maximum 100 mA). A timer was started to determine how quickly the symptoms disappeared after TENS initiation. The treadmill stress test was classified as TENS positive, dubious or negative. A TENS-positive test, i.e. resolution of angina pectoris more quickly than if short-acting nitroglycerin (NTG) was used, was an indication for implantation of a spinal cord stimulator. If the test was TENS dubious, i.e. resolution of angina pectoris as quickly as with the use of short-acting NTG, or TENS negative, i.e. no angina pectoris during the treadmill stress test, TENS was continued during 1 month and evaluated. If the patient benefitted from TENS after 1 month of use either implantation of a spinal cord stimulator or continuation with TENS were treatment options, depending on the wish of the patient. If the patient derived no benefit from TENS after 1 month treatment was stopped. During the initial visit the same team consisting of two interventional cardiologists, anaesthesiologist/pain specialist, physiotherapist and specialist pain nurse was present to evaluate the anamnesis and the results of the treadmill stress test performed by the patient.

### Implantation of SCS device

Implantation of the SCS device is performed by the anaesthesiologist/pain specialist with the patient under sedation. Using a percutaneous approach the epidural compartment is accessed in the thoracic region. The lead is placed in the epidural space with the active part situated in the higher thoracic and lower cervical segments. The correct position of the lead is determined, with the sedation temporarily reduced, by using stimulations and the patient response. At the epidural entry point the lead is fixated and attached to an implantable pulse generator in the left buttock via a subcutaneous route. Both the entry site of the lead as well as the pulse generator pocket are closed, finishing the implantation procedure. In general the patients are able to leave the hospital the next day.

### Endpoints

#### Primary endpoints

Quality of life and frequency of angina symptoms were the primary endpoints in this study. From patients who underwent SCS, data were collected using a general questionnaire (RAND 36-Item Health Survey [RAND-36]) and a disease-specific questionnaire (Seattle Angina Questionnaire [SAQ]). Patients were asked to complete these questionnaires at baseline, prior to implantation of the spinal cord stimulator, and 1 year after implantation. Both questionnaires have been validated for evaluating quality of life and the SAQ for evaluating severity of angina pectoris in patients with RAP and both have been used in other studies ([7, 10, 11]; Electronic Supplementary Material, Appendix 1). These questionnaires cannot be directly compared to each other since the SAQ is disease specific and the RAND-36 is a general health questionnaire.

#### Secondary endpoints

The secondary endpoints were use of short-acting nitrates (data collected from the SAQ) and clinical endpoints, including admission to the hospital due to chest pain and/or non-ST-elevation myocardial infarction (NSTEMI, defined as high sensitivity troponin T with at least one value above the 99th percentile and/or new ischaemic ECG changes and/or development of pathological Q waves), PCI due to NSTEMI or progressive stable angina pectoris at outpatient clinic follow-up, cardiovascular mortality and all-cause mortality during the follow-up period of 1 year. The last secondary endpoint were complications after the implantation of the spinal cord stimulator such as infection, lead and/or battery repositioning at 30 days and after 1‑year follow-up. The data were collected by reviewing the patient files.

### Statistical analysis

To analyse the results from the SAQ and RAND-36 questionnaires the paired Student’s *t*-test was used. If the values were not normally distributed the Wilcoxon signed rank test was applied. Analysis was performed when both the baseline and 1‑year data were available from the same patient. A probability value of <0.05 was considered statistically significant. All analysis was performed using SPSS version 23.0 for MacBook (SPPS, Inc., Chicago, IL, USA).

## Results

### Patient selection

**Fig. 1:**
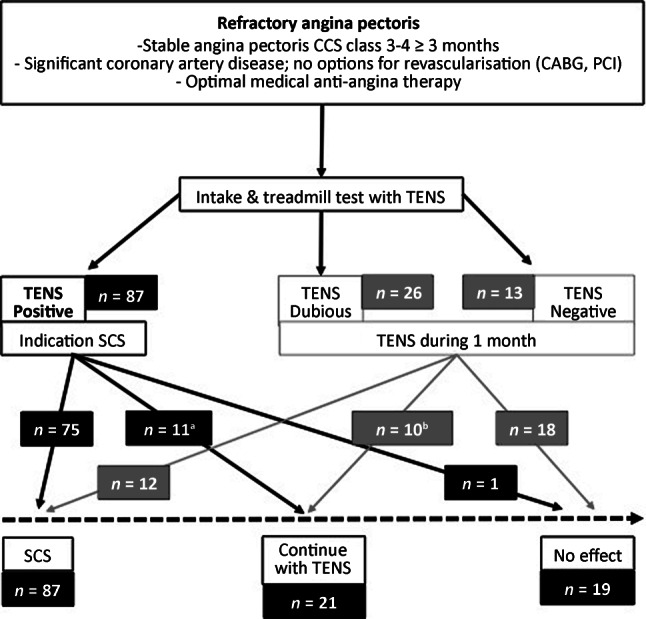
Selection process. (*CCS* Canadian Cardiovascular Society, *CABG* coronary artery bypass graft, *PCI* percutaneous coronary intervention, *TENS* transcutaneous electrical nerve stimulation, *SCS* spinal cord stimulation. ^a^Reasons for TENS instead of SCS: patient request (*n* = 4), comorbidities (*n* = 5), implantation of SCS device technically unsuccessful (*n* = 2). ^b^This group of ten patients includes one patient who did not perform a treadmill stress test due to a lower leg amputation but received TENS during 1 month and continued with this treatment option)

From July 2010 through to March 2017, 127 patients who met the inclusion criteria for RAP were referred to our hospital. During the initial visit 126 patients performed a treadmill stress test. Of those, 87 patients had a TENS-positive test and therefore an indication for implantation of a spinal cord stimulator. Twenty-six patients had a TENS-dubious test and 13 patients a TENS-negative test with an indication for continuation of TENS during 1 month. A final number of 87 patients received SCS, 21 patients continued with TENS and 19 patients had no benefit and withdrew from treatment. In the group with a TENS-dubious or TENS-negative test, 12 patients crossed over to SCS due to the beneficial effect of TENS during the 1‑month trial period. Ten patients continued with TENS and 18 patients reported no symptom relief using TENS and stopped treatment. In the group with a TENS-positive test, 11 patients crossed over to the final treatment option of continuing with TENS instead of SCS for various reasons. Four patients declined implantation of a spinal cord stimulator, opting to continue treatment with TENS. Six patients had comorbidities with a contra-indication for implantation of a spinal cord stimulator, and it was decided to continue treatment with TENS. During follow-up one of these six patients did not find TENS beneficial and withdrew from treatment. In two patients implantation of a spinal cord stimulator was unsuccessful, with inadequate paraesthesias in the right location during positioning of the epidural lead; these patients continued treatment with TENS (Fig. 1). One patient could not perform a treadmill test due to a lower leg amputation and received TENS with good effect.

### Patient characteristics

In total 87 patients received an SCS device. Their average age was 63.8 years and 88.5% were male. Ninety-two percent had angina pectoris CCS class III or IV. Ischaemia was proven by MIBI-SPECT in 69%.

**Table 1:** Baseline patient characteristics

	Spinal cord stimulation (*n* = 87)
Age, years (mean ± SD)	63.8 (±9.09)
Male (%)	88.5
CCS class III or IV (%)	92.0
*Risk factors (%)*	
Positive family history of CVD	52.9
Active smoker	13.8
Hypertension	95.4
Hypercholesterolaemia	97.7
Diabetes	39.1
Myocardial infarction	62.1
*Previous interventions (%)*	
PCI	83.9
CABG	82.8
*Medication use (%)*	
Acetylsalicylic acid	85.1
P2Y_12_ inhibitor	66.7
OAC	16.0
Beta blocker	85.1
Dihydropyridines	55.2
Non-dihydropyridines	11.5
Ivabradine	12.6
ACE or ARB inhibitor	71.3
Diuretics	29.9
Statins	94.3
NTG short-acting	86.2
NTG long-acting	81.6
Spironolactone	12.6
*Left ventricular ejection fraction (%)*	
Normal (>50%)	82.8
Impaired (35–50%)	11.5
Reduced (<35%)	3.4
*Miscellaneous (%)*	
PM/ICD	10.3
Ischaemia	69.0

*CCS* Canadian Cardiovascular Society, *CVD* cardiovascular disease, *PCI* percutaneous coronary intervention, *CABG* coronary artery bypass graft, *OAC* oral anticoagulants, *ACE* angiotensin-converting enzyme, *ARB* angiotensin II receptor blockers, *NTG* nitroglycerin, *PM* pacemaker, *ICD* internal cardiac defibrillator

### Primary endpoints

#### Seattle Angina Questionnaire

**Fig. 2:**
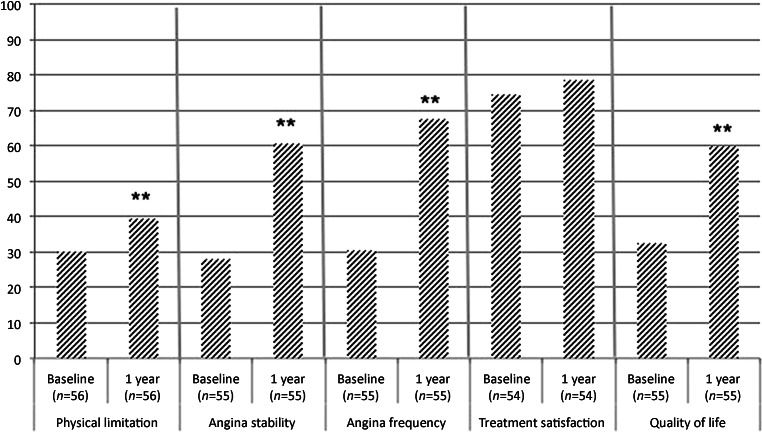
Results of Seattle Angina Questionnaire at baseline versus 1 year. (**p < 0.001)

A total of 56 (64.4%) patients completed the questionnaire at baseline and at 1 year. One-year follow-up showed a statistically significant improvement in four of the five dimensions of the SAQ. The improvement in satisfaction with treatment was not statistically significant (*p* = 0.55) (Fig. 2).

#### RAND-36 questionnaire

**Fig. 3:**
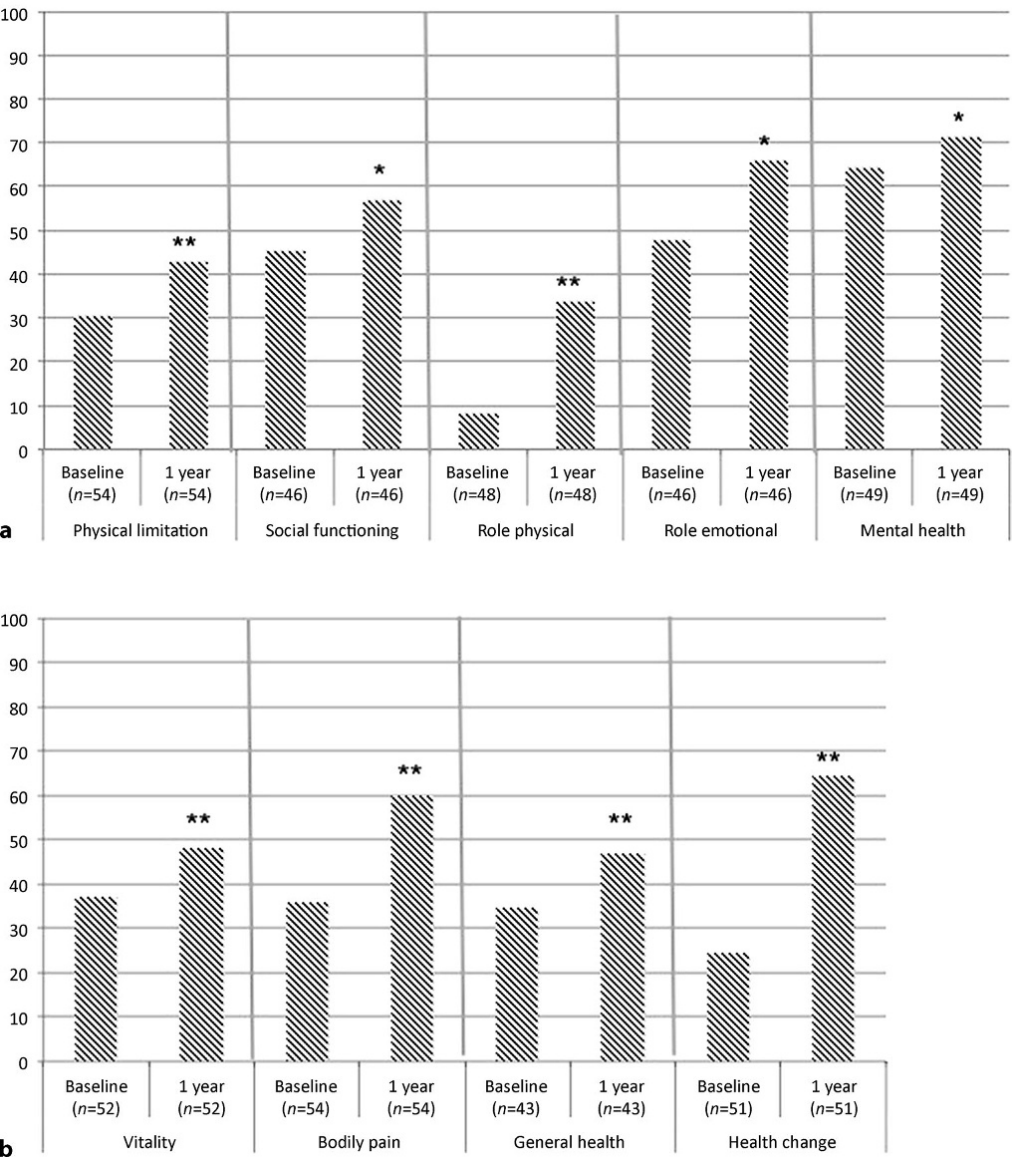
**a,** **b** Results of RAND 36-Item Health Survey at baseline versus 1 year. (*p < 0.05, **p < 0.001)

A total of 54 (62.1%) patients completed the questionnaire at baseline and at 1 year. One-year follow-up showed a statistically significant improvement in all nine dimensions of the RAND-36 (Fig. 3).

#### Frequency of angina pectoris

**Fig. 4:**
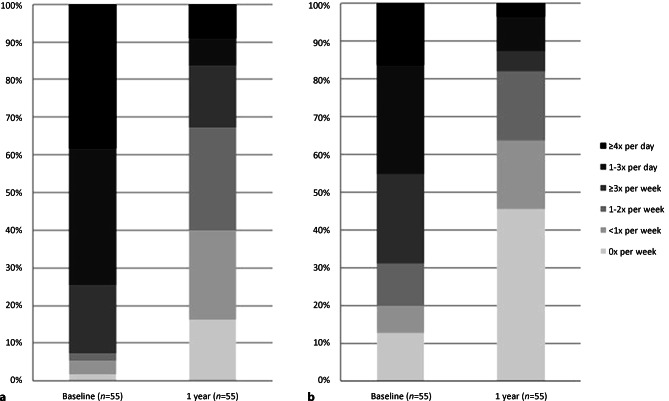
One-year results of frequency of angina pectoris (**a**) and frequency of use of short-acting nitrates (**b**)

The frequency of angina pectoris attacks had decreased significantly from more than 4 times a day to 1–2 times a week at 1‑year follow-up (*p* < 0.001) (Fig. 4a).

### Secondary endpoints

#### Use of short-acting nitrates

The use of short-acting nitrates due to angina pectoris had decreased significantly from 1–3 times a day to less than once a week at 1‑year follow-up (*p* < 0.001) (Fig. 4b).

#### Clinical endpoints

During the follow-up period of 1 year a total of 16 patients were admitted to hospital due to chest pain, eight patients due to a NSTEMI and eight patients due to a non-cardiac cause of chest pain. Of the eight patients admitted to hospital due to a NSTEMI, four underwent a PCI and four were treated conservatively. Another six patients underwent PCI due to progressive angina pectoris during outpatient follow-up. Three patients died during the follow-up period, one of them due to a cardiovascular cause (Tab. 2).

**Table 2:** Clinical endpoints

Clinical endpoints	Spinal cord stimulation (*n* = 87)
*Hospital admissions for chest pain*	16 (18.4)
NSTEMI	8 (9.2)
Non-cardiac cause	8 (9.2)
*Percutaneous coronary intervention*	10 (11.5)
NSTEMI	4 (4.6)
Stable angina pectoris	6 (6.9)
*All-cause mortality*	3 (3.4)
Cardiovascular mortality	1 (1.1)

Values are *n* (%)

*NSTEMI* non-ST-elevation myocardial infarction

#### Implantation of SCS device

At 30 days after implantation of a SCS device no complications had occurred. During 1 year of follow-up two patients had to undergo repositioning of the epidural lead. During the follow-up period of 1 year no infections occurred and there was no need for battery repositioning.

## Discussion

The main findings of this study were a statistically significant improvement in the quality of life and a statistically significant reduction in the frequency of angina pectoris from more than 4 times a day to only 1–2 times a week in patients with RAP being treated with SCS during 1 year of follow-up. Secondary findings of this study were a significant reduction in the use of short-acting NTG use from 1–3 times a day to less than once a week, low cardiovascular mortality (1.1%) and low all-cause mortality (3.4%).

SCS was indicated in 89 patients and a device was successfully implanted in 97.8% (*n* = 87) of these patients. In only two patients was the implantation not successful, and these patients continued with TENS. The implantation procedure was safe with no complications occurring within 30 days of the implantation. During 1 year of follow-up two patients had to undergo a revision of the lead position.

This study was a large, single-centre, prospective observational study. All patients referred to our centre for treatment of RAP were evaluated by the same team. This team included two interventional cardiologists, an anaesthesiologist/pain specialist, a physiotherapist and a specialist pain nurse. A standardised treadmill stress test was used to induce angina pectoris. The same TENS system with standardised settings was applied to treat the induced angina pectoris, and the team determined whether the test was positive, dubious or negative. Selection bias was minimised by protocolled screening and a symptom-inducing treadmill test executed by a multidisciplinary RAP team. This is in contrast to the most recent and largest study by Andrell et al. (from 2010), who included 121 patients from ten different centres [7]. In their study screening and selection process varied with each site, leading to possible selection bias.

The questionnaires used in the current study to evaluate quality of life and angina pectoris symptoms were the RAND-36 (general) and SAQ (disease specific). Although the percentages of complete follow-up of SAQ and RAND-36 (64.4 and 62.1% respectively) seem low, they are in line with those in the literature [12, 13]. All dimensions showed a statistically significant improvement at follow-up, with the exception of the dimension ‘treatment satisfaction’ of the SAQ, which encompasses how patients feel about their treatment in general and not specifically about the SCS treatment. The conclusion was that patients were already satisfied with the treatment they were receiving prior to being treated with SCS, leading to no significant improvement, and is in line with previous studies [3, 8, 14, 15].

One of the criteria in the definition of RAP was optimal anti-angina medication. Of our study population, 85.1% used beta blockers, 66.7% calcium antagonists, 81.6% long-acting and 86.2% short-acting nitrates. As many as 89.7% of the patients used at least two types of anti-angina medication (beta blocker, calcium antagonist and/or long-acting nitrate) and 48.3% used three types, confirming that optimal medical therapy was being received. This is comparable with other studies in patients with RAP.

In this study we included a high percentage of patients (69%) with ischaemia, as proven by MIBI-SPECT, which is important to avoid possible bias from patients with non-cardiac chest pain. Several RAP studies used reversible ischaemia (proven by MIBI-SPECT, ST-segment depression >0.1 mm during 48‑h ambulatory ECG monitoring or exercise testing) as an inclusion criterion to recruit patients. However, the majority of these studies did not report the actual percentage of proven reversible ischaemia in the baseline data [3, 14–19]. Of those studies that did report the percentage of proven ischaemia, in the most recent and largest study by Andrell et al. it was 57% [7]. In this study subanalysis showed no difference as regards quality of life or angina pectoris between patients with and without proven ischaemia on MIBI-SPECT.

This was an open, prospective observational study, meaning that there was no control group. However, previous randomised controlled trials (RCTs) have had problems including sufficient patients, leading to small samples varying from 12 to 32 patients, specifically the two studies with a true control placebo group [8, 14]. There have been several studies comparing SCS treatment with other treatment options for RAP, but this does not represent a true control group. Also with SCS treatment it is difficult to perform a double-blind study because the patient can feel if the stimulations are present or not. To date the majority of the data with regard to the effect of SCS in patients with RAP has been provided by prospective observational studies and meta-analysis, which are important because of the difficulties in performing a methodologically sound RCT. The prospective observational studies that have been performed have shown a beneficial effect in reducing angina symptoms and improving quality of life. This includes the current prospective observational study, which is an important addition to the current knowledge of the effects of SCS because clear inclusion criteria and a uniform selection process by the same multidisciplinary team were used.

In this single-centre, prospective, single-arm observational study, involving a multidisciplinary team in the selection process, SCS treatment in patients with RAP significantly improved the quality of life during 1 year of follow-up. Furthermore, the frequency of angina pectoris episodes significantly decreased.

## Caption Electronic Supplementary Material

Example of the scoring system used in the Seattle Angina Questionnaire
