# Supplementary material for: Improvement in quality of life and angina pectoris: 1-year follow-up of patients with refractory angina pectoris and spinal cord stimulation
Source: Neth Heart J. 2020 May 19;28(9):478–84. doi: 10.1007/s12471-020-01422-0 (PMC7431482; doi:10.1007/s12471-020-01422-0)
Supplement: Supplementary file 1 — Example of the scoring system used in the Seattle Angina Questionnaire [file 12471_2020_1422_MOESM1_ESM.docx]

**Appendix (Electronic Supplementary Material)**

Appendix 1:

Seattle Angina Questionnaire (SAQ). Question 1a through to 1i is used for the dimension physical limitation. For each question a value of 1 up to 6 can be scored, with a maximum score of 54.

Example: if a patient has filled in “somewhat limited” for question 1a through to 1i, a score of 3 is given for each answer. With a total score of 27. Using the previously mentioned formula: $\frac{(\mathbf{value}-lowest possible value per dimension)}{range value per dimension}$ X100, (lowest possible value = 9, range value = 45), $\frac{(\mathbf{27}-9)}{45}$ X100 a value of 40 is calculated for the dimension physical limitation.
